# The Dutch East India Company through the Local Lens: Exploring the Dynamics of Indo-Dutch Relations in Seventeenth Century Bengal

**DOI:** 10.1177/0376983617712811

**Published:** 2017-06-30

**Authors:** Byapti Sur

**Affiliations:** 1 Institute of History, Leiden University, The Netherlands

**Keywords:** Dutch East India Company, Bengal, *Mangalkavya*, Mughal, seventeenth century, Indian Ocean

The Dutch East India Company (VOC) shared a history of two hundred years of coexistence with the locals in Bengal. And yet their official reports had little to say about this relation, except frequent complaints against the locals and the accompanying, inherent distrust. There has been, however, a significant amount of historiography that has developed in the recent decades on Indo-Dutch contacts based on the information available in the sources. This article aims to add more nuances to these dynamics, by showing how the Company and its officials were seen by the locals in Bengal. It argues that the local–Dutch relation had not just been about static characterisations of ‘partnership’, ‘cooperation’ or ‘conflict’, but was rather dependant on personal networks and profit motives backed by diverse social positions. The Dutch in the perception of the locals had different meanings, images and implications. Through the study of three objects—local texts, a Dutch painting and a legal case—this article aims to capture precisely these very perceptions in contributing towards the complex of Indo-Dutch interactions in seventeenth century Bengal.

Indian Historical Review
44(1) 62–91
© 2017 ICHR
SAGE Publications
sagepub.in/home.nav
DOI: 10.1177/0376983617712811
http://ihr.sagepub.com

**Byapti Sur**[1]

The Dutch East India Company (VOC) shared a history of two hundred years of coexistence with the locals in Bengal. And yet their official reports had little to say about this relation, except frequent complaints against the locals and the accompanying, inherent distrust. There has been, however, a significant amount of historiography that has developed in the recent decades on Indo-Dutch contacts based on the information available in the sources. This article aims to add more nuances to these dynamics, by showing how the Company and its officials were seen by the locals in Bengal. It argues that the local–Dutch relation had not just been about static characterisations of 'partnership', 'cooperation' or 'conflict', but was rather dependant on personal networks and profit motives backed by diverse social positions. The Dutch in the perception of the locals had different meanings, images and implications. Through the study of three objects—local texts, a Dutch painting and a legal case—this article aims to capture precisely these very perceptions in contributing towards the complex of Indo-Dutch interactions in seventeenth century Bengal.

> I had a book in my hands to while away the time, and it occurred to me that in a way a landscape is not unlike a book—a compilation of pages that overlap without any two ever being the same. People open the book according to their taste and training, their memories and desires: for a geologist the compilation opens at one page, for a boatman at another, and still

> another for a ship's pilot, a painter and so on. On occasion these pages are ruled with lines that are invisible to some people, while being for others as real, as charged and as volatile as high-voltage cables.
>
> – Amitav Ghosh, *The Hungry Tide*[2]

The state of West Bengal, lying today in the eastern coast of India had been a larger province in the seventeenth century including modern Bangladesh, parts of Orissa, Assam, Bihar and Tripura in India. After being conquered and subjected to the Mughal administration, this amalgamation of regions came to be labelled under the single entity of *Subah Bangal* (the Mughal provincial unit for administering Bengal).[3] Bordering the north of the Bay sharing its namesake [Bay of Bengal], it has undergone several geographical changes throughout time.[4] Battered and blown by numerous consequent shifts in political forces, Bengal witnessed violent moments like that of the partition or the intermittent losses of various cities and towns. But for centuries, its riverine networks continued to serve as a nodal point in traversing the Indian Ocean as well as connecting it further inland. Even after the building of railways in the nineteenth century, Tapan Ray Chaudhuri exclaimed at the ease with which his grandfather could reach the small village of Kirtipasha (now in modern-day Bangladesh) directly from Agra by water, carrying heavy and fragile goods like marble craftworks for his personal collection.[5] At the same time, a lot of revenue was realised from the cultivation here, as is evident from the *A'in-i-Akbari* that led to a growing prosperity of the Bengali zamindars under the colonial regime.[6] Bengal's uniqueness therefore lay in its 'amphibious' character—its rich agricultural soil made fertile by the silt brought by its rivers whose fresh waters meandered their way into the salty vastness of the sea.[7] Travellers, traders, warriors, boatmen, pilgrims, poets and painters from China, Persia, Africa, Portugal and many more territories visited this region and its waters at different points of time and recorded their impressions and encounters which went on to make Bengal's history. All these experiences were personal perceptions and individual accounts that, as Ghosh expresses in the lines quoted above, presented a spectrum of different realities and images of one Bengal.

When the Dutch East India Company (VOC) arrived here in the early years of the seventeenth century, they too wrote down about their encounters with the locals. An entire archive of official reports, however, has left us with a largely one-sided story with very little of the local voices to be heard or told. Only moments of discontent and conflict brought the locals to the fore in the VOC reports, stereotyped as 'ill-mannered' and 'submissive' subjects of a tyrannical and despotic Mughal ruler.[8] The huge amount of literature produced in the recent years have tried to capture extensively the dynamics of these Indo-European relations. From the 'Vasco da Gama

[2] Ghosh, *The Hungry Tide*, p. 444.
[3] Abu'l-Fazl, *Ain-I Akbari*, trans. H. Blochmann, Vol. II (Delhi), p. 133.
[4] Bhattacharya, *Historical Geography*; Eaton, *The Rise of Islam*, pp. xxiii–xxiv; Mukherjee, *Strange Riches*, pp. 90–158.
[5] Chaudhuri, 'Bangalnama', p. 40.
[6] Abu'l-Fazl, *Ain*, Vol. II, pp. 143–54.
[7] Mukherjee and Seshan, 'Introduction', pp. 147–49.
[8] The VOC sources are littered with such stereotypes. See Van Dam, *Beschryvinge*, Vol. II, Part 2, p. 18.

epoch' to the 'Age of Partnership', from the 'Age of Commerce' to the 'Age of Contained Conflict'; debates on the local–European relations have expanded and evolved in all directions.[9] But barring a few commendable works produced in the last decades, there has not yet been enough research done on bringing out the converse perspective of the locals about the Europeans.[10] The story of the VOC in Bengal has therefore also been no exception. One is left to wonder if there is at all any way to bring back the local voices that have been doomed to remain unheard of. How can the local perceptions and their relations with the Company servants be unravelled for seventeenth century Bengal? Is it possible to restore properly the local agency in these hundred years of the Company's existence that the official reports have stripped them of? I believe that there is a way and it is through the exploration of local mentalities at various levels of the society that their perceptions about the Dutch can be developed. This essay is a humble attempt along these lines to excavate the local voices by revisiting the dynamics of Indo-Dutch relations in Bengal. It promises to review the old landscape through a new window: the VOC in Bengal as seen through the eyes of the locals.

To trap the perceptions of the different ranks of locals, three objects have been used representing their diverse social backgrounds—namely *Mangalkavya* literature, a Dutch painting depicting the locals and a legal case involving a Bengali broker. The textual evidence at hand, if I must admit, happen to form quite a flimsy layer of non-European sources. The other two VOC sources are, however, used according to the traditional method of deciphering information by sometimes reading along and sometimes reading against the grain. Woven together into a composite whole, they tell a story of the Company servants living and working together with the local communities in Bengal. It of course remains told from the local viewpoint. In the process, it reveals that the blanket assertions of 'cooperation', 'partnership', 'conflict' or such other specific terms in characterising local–European relations do not hold water. Instead, it argues, that different social positions and personal networks determined whether a relation turned out to be harmonious or hostile. Together they help in getting the locals' insight into the seventeenth century Dutch lives.

## Local Texts

The local texts produced in seventeenth century Bengal and written in the Bengali language belongs largely to the genre of *Mangalkavya* literature.[11] *Mangal* means blessings or prosperity and *kavya* means text. Thus, these texts were meant to bring in spiritual goodness and prosperity when one read or listened to them. They were didactic, religious poems composed by poets under the patronage of small local *rajas* (kings) affiliated to the Brahmanic faith. The practice of disseminating the contents of

[9] See for a brief overview of the historiography Vink, 'Indian Ocean', pp. 41–62; Subrahmanyam, *Explorations in Connected History*; Kling and Pearson (eds), *The Age of Partnership;* Furber, 'Asia and the West', pp. 711–21; Reid, *Southeast Asia.*

[10] Flores, 'Floating Franks', pp. 33–45; Subrahmanyam, 'On the Hat-Wearers', pp. 45–81; 'Taking Stock', pp. 69–100.

[11] For more details on *Mangalkavya* see Chatterjee, *Cultures of History*, pp. 90–117; Sen, *Bangla Shahityer Itihash*, p. 97; *Banglapedia* http://en.banglapedia.org/index.php?title=Mangalkavya the *Mangalkavya* literature was to read them aloud in public as a form of oral performance or verbal play, sometimes accompanied by music for its listeners.[12] These poems were aimed at celebrating the worship of lower non-Brahmanic deities like Manasha (the venomous snake-goddess), Chandi (the wild, dark-skinned, warrior goddess) and Dharma (the god of righteousness and virtuous rule). The protagonists of these poems were primarily local kings and wealthy merchants who were shown to have accepted these deities as part of their household gods and adhere religiously to their accompanying worship rituals. The plot revolved either around the ways in which these lower gods compelled the protagonists to accept their authority as a household deity or the ways in which some of these deities were themselves reborn in their human incarnations to assert their position on earth. Even though the *Mangalkavya* poems were thought to bring 'domestic bliss and happiness', their performance did not form an indispensable part of the religious ceremonies in Bengal.[13]

The question that then arises concerns the historical validity of these poems and the authenticity of their information in highlighting perceptions of the locals about the Europeans. To find answers, one needs to know why these texts were produced in the first place. Historians of medieval Bengali literature argue that the *Mangalkavya* literature emerged in Bengal from the fifteenth century onwards as a Brahmanic reaction to the emergence of Islam and other sects like Vaishnavism.[14] After uprooting the Sena rulers who were fervent Brahmanic followers; the Afghans, the Mughals and the other Muslim dynasties ruled in Bengal till the eighteenth century.[15] It was during this time that Vedic Brahmanism began to lose its social base and authority among the ordinary people of this land. The smooth adaptation of the local language and culture by the Muslim rulers of Bengal (the Husain Shahi dynasty was particularly remarkable in this context) led to the creation of not only a significant Muslim population, but also a strong syncretic culture fostering the rise of the Vaishnava, Sahajiya and Sufi cults which alienated more people from the Brahmanic dominion.[16] The non-Brahmins, tribal groups and other outcastes were now offered a multitude of options including Islam and these other dissenting sects.

The Brahmins therefore lost no time in investing a considerable amount of energy behind maintaining their fast eroding subject base with the help of the small rajas as their patrons. In light of Kumkum Chatterjee's argument that the Brahmins in Bengal enjoyed less of a privileged position unlike their South Indian counterparts, the composition of these texts as a desperate attempt to reinstate lost glory makes all the more sense.[17] The marginal sections of the society, chiefly the tribal groups, the unsettled population of the jungles and other lower castes were the primary targets of this endeavour as they were the ones who came to be associated with nature worship. The active incorporation of deities like Manasha or Chandi representing the snake

[12] Curley, 'The 'World of the Text', p. 185; Sen, *Bangla Shahityer Itihash*, pp. 198–99.
[13] Chatterjee, *Cultures of History*, pp. 90–91.
[14] Ibid., p. 92; Sen, *Bangla Shahityer Itihash*, p. 98.
[15] Sen, *Eastern Bengal Ballads*, p. xxvi.
[16] On Islam in Bengal, see Eaton, *The Rise of Islam*; Dasgupta, 'Islam in Bengal', pp. 30–41; Chatterjee, *Cultures of History*.
[17] Chatterjee, 'Scribal Elites', p. 451.

and other wild elements into the Brahmanic fold could be justified in this connection. Most of these *Mangal* poems, as Curley suggests, highlighted the contrast between the two frontiers of Bengal—the Western peneplain below the Chota Nagpur plateau and the lower delta in the southeast indicating the worlds of the 'forest' versus the 'settled domain', respectively.[18] With the coming of new political rulers in the scenario, alternative religious and ethnic forces made inroads. The nomadic people started pushing their frontier and gradually settling down, thanks to the land grants boosted by Muslim governors and rulers.[19] It was during this time that they chose to embrace easier religious options like a more flexible Islam over the rigid Vedic and Brahmanic dogmas. This made Brahmanism realise that it had to either die out or change its framework for accommodating these new settlers in the changing ambience.

The *Mangal* texts could therefore be seen as reactions of the older powerholders to retain their power structures in the face of new political challenges. It is a misunderstanding however to assume that the contents of these texts were necessarily hostile or aggressive or anti-Islamic. Chatterjee's work reveals how they had a moderate tone and were accommodative to attract even the Afghan Muslim rulers and the Mughal governors within the Brahmanic fold.[20] Nevertheless, most scholars accept that the authors of the *Mangal* texts with both Sanskrit and Persian education had a hard time trying to strike an uneasy balance between the Brahmin kings as their patrons and their Mughal overlords.[21]

To come back to its historical validity therefore, one may compare them to the modern-day genre of historical fictions.[22] The landscape, the wars, the social scenario, the cultural practices and the economic effects could possibly be historically traced and put into context. Thus, the setting of the stories and the social problems they addressed were most of the times historically valid though all the minor details were not always in the right place. But the characters and the causation of events lay in gods and monsters which gave that fictional tinge to these texts. They manifested a liminality between historical reality and fiction. To borrow Curley's words, 'in *mangal-kavya* a partial intersection between fictional and actual worlds was made possible by interweaving realistic and fabulous modes of representation, and so inviting both more literal, particular, and distant, and more symbolic and immediate interpretations'.[23]

It is in these texts that the Dutch appeared for the first time to be mentioned as a group of Europeans in the eighteenth century. It occurred in Bharatchandra Ray's *Annadamangal* that was patronised by Maharaja Krishnachandra in Nadia.[24] Ray however placed the plot of his story in the previous century. He depicted the city of

[18] Curley, 'Kings and Commerce', p. 302; Wink, *Al-Hind*, Vol. III, p. 162.
[19] Dasgupta, 'Islam in Bengal', pp. 33–35.
[20] Chatterjee, 'Goddess Encounters', pp. 1435–87.
[21] Chatterjee, 'Scribal Elites', p. 464; Curley, 'Maharaja Krsnacandra', *Poetry and History*, pp. 4, 11–15.
[22] See for more details on historical analysis, Curley, 'A Historian's Introduction', *Poetry and History*, pp. 1–70.
[23] Curley, 'World', p. 186.
[24] Bharatchandra Ray's family was patronised by the Mughals and he was proficient in Persian as well. But his direct patron was Maharaja Krishnachandra of Nadia whose family was also known to have received royal favours from the Mughals and worked for them.

See *Banglapedia* for more details, http://en.banglapedia.org/index.php?title=Bharatchandra_Ray

Gaur (the political capital of Bengal) and its various quarters of residence through the eyes of the Mughal *subahdar* (the Mughal noble in charge of a subah), Man Singh who had been dispatched by Emperor Jahangir to crush the rebellions of the *bara bhuiyans* (twelve landlords).[25] The author described Man Singh as moving ahead into the city from one quarter of residence to another and on coming across the first quarter, he saw an European mélange. The following description was given:

> In the first quarter (of the city of Gaur) lives the hat-wearers,
> The Ingrej (English), the Olondaj (Dutch), the Firingi (Portuguese), the Farash (French);
> The Dinemar (Danish) and the Eleman (from Alemania) who fire with their cannons,
> They sail and bring different commodities loaded on their ships.*[26]

This was the first explicitly written evidence about the local perceptions of the Dutch. What could it have meant? Ray's description points to a stereotypical image of the powerful merchant-warrior type of Europeans. Based on it, one can assume that the locals saw them as foreigners with a distinct style of dressing and as powerful men with commercial and military prowess. Can it then be concluded that the locals, representing here the intermediate ruling groups as patrons of these texts, viewed the Dutch as powerful overlords? Considering that the text was composed in the eighteenth century about a seventeenth century event, it is highly likely that the author imposed his eighteenth-century observations on a seventeenth century incident. But what seems to be apparent from this piece at first glance is that the locals, as small kings and zamindars, viewed the Dutch with some amount of awe and fear.

Such clichéd depictions of power and dominance were attributed, however, not only to the Dutch in particular, but also to the Portuguese who had been there before. Similar images of the mighty and the dreaded *firangi* pirates, implying the Portuguese appeared repeatedly in these poems. Kabikankan Mukundaram in *Chandimangal* talks about the threat of the '*firangi harmad*' in the waters of the Bay of Bengal that had been a reason for fear for many local merchants.[27] Besides that, a few Mughal chronicles like the Shah Jahan Nama and the *Baharisthan-i-Ghaibi* also expressed their prejudice against the *firangis.*[28] Subrahmanyam points out how certain texts from other regions written in Persian and Malayalam echoed these stereotypical ideas of the Portuguese as 'deceitful' and 'religious bigot(s)'.[29]

[25] Man Singh was known to serve Akbar as the *subahdar* of Bengal from (1594–1605) and later for a year under Jahangir (1605–1606).
[26] Ray, 'Annadamangal', Vol. II, p. 10.
* I am grateful to Professor Sudipta Sen for helping me with the translation of certain parts in this text.
[27] The word *Harmad* is a distortion of the word *armada.* See Mukundaram, *Kabikankan Chandi*, p. 126.
[28] Begley and Desai, *The Shah Jahan Nama*, pp. 85, 117 (here Christian prisoners from Bengal are referred to as 'icons of these infidels'), 249–51. For the phrase '*farangian-i-harmad*', see Nathan, *Baharisthan*, pp. 86, 816.
[29] Subrahmanyam, 'Taking Stock', p. 96.

However, a closer analysis of Ray's *Annadamangal* might help in busting such stereotypical perceptions. If seen against the political conditions that his patron Raja Krishnachandra underwent, the reason for such depictions of the Dutch and the Europeans can be explained. Raja Krishnachandra, the ruler of Nadia hailed from a Brahmin family that had sought royal favours with the Mughals and become zamindars, thus not being unfamiliar to the Persian courtly culture.[30] At the same time, they were also in touch with the Dutch, the English and the other Europeans that traded in the neighbouring areas. This was because Nadia, that lay along the banks of the river Hugli, happened to be located in the middle of the route connecting Calcutta with Kasimbazaar and Malda.[31] While Calcutta was the English base in the eighteenth century, the factories of the Dutch had been there throughout the seventeenth century in Malda and Kasimbazaar.[32] These places with the European companies received regular supplies of textiles, especially muslin from Shantipur, in Nadia.[33] Besides this, there were moments when members of the royal family established financial connections with the European companies as had been the case of Raja Ramkrishna who gave money to the English in 1697 in return for military help to crush the rebellion of Shobha Singh in Bengal.[34] After the Battle of Plassey when the English had amassed enough power to intervene in the political organisation of Bengal, the revenues of Nadia were assigned to them by Mir Jafar. This erupted tensions between Raja Krishnachandra and the English.[35] Such relations of dependency for political legitimacy on the Europeans to whom the ruler of Nadia had to pay his revenues and his helplessness before a larger military force with greater economic resources, explains for the visions of the Europeans in Ray's *Annadamangal*.

What is worth noting, however, is the relative absence of great details about the Dutch in many of the seventeenth century *Mangalkavya* literature. It is quite strange to think that the composers of these texts hardly felt it necessary to record their impressions about the Dutch despite the latter's considerable presence in Bengal. The answer lies in seeing the situation in Bengal in the seventeenth century with a still strong Mughal control as being essentially quite different from the eighteenth. The authors of these *Mangal* texts and the small kings, as their patrons, referred to only those people in their compositions who mattered for their survival like their Mughal overlords. Thus, even though these local rulers had contact with the Europeans, they did not necessarily consider them important enough to be mentioned in their poems while the Mughals received generous attention. It leads us to think that they definitely till then did not see the Dutch or any other European group as potential alternatives for seeking legitimacy for their small kingdoms. This was a far cry from the scenario at the end of the eighteenth century when there were hints of the European Companies, including the Dutch, being seen as potential powers in the political sphere of the subcontinent.

[30] Curley, 'Maharaja Krsnacandra', *Poetry and History*, p. 1.
[31] Ibid., p. 2.
[32] NA, NL-Ha, VOC, 1.04.02, OBP, inv. nr. 1421, Letter from Van Reede to the *Heren XVII* from Hooghly, Bengal dated 9th December, 1686, f. 91v.-96r.
[33] Curley, 'Maharaja Krsnacandra', *Poetry and History*, p. 2.
[34] Ibid., p. 2.
[35] Ibid., pp. 40–43.

When the following lines commonly attributed to the *baul* (a sect in Bengal) singer Lalon Fakir were sang in the nineteenth century, the Europeans had already become serious political contenders next to their Indian competitors:

> Oh my Magh, Firangi, Olondaj,
> Hindu and *Mussalman* brothers
> There is one God who is the creator of all matter
> And thus everybody is equal and one.[36]

These words were meant to appeal to all those major groups who had been striving for political eminence at that time in Bengal. The Dutch along with the other Europeans had by the nineteenth century made a mark as potential powerholders for the local intermediate landlords and *rajas*.

The relation between these local elites and the Dutch could thus be characterised as a loose and manipulative encounter in the seventeenth century, common to kinglets dealing with foreign merchants. Curley points out similar behaviour of the king of Sri Lanka towards the merchant Dhanapti in Mukundaram's *Chandimangal*.[37] It was when Dhanapati, sent by the king of Gaur to fetch certain commodities from Sri Lanka, justified himself as a foreign (*bideshi*) trader before the king of Sri Lanka that the latter's guarded and suspicious nature came forth.[38] Curley thus wrote:

> By definition foreign merchants do not belong to the kingdom in which they appear as strangers, and, according to Mukunda's poem, they should not establish a permanent residence therein. They carry both wealth and weapons. Reactions to them accordingly are ambivalent: they are both desired and feared. In all these respects they are liminal. Their state of 'not belonging' makes foreign merchants more vulnerable to the king in whose land they are strangers… When merchants appear in a foreign king's port, they may advertise the martial capacity which their own king has given them as his servants, and they may in fact be his spies or thieves sent to pillage the foreign king's land. At the same time, they carry great wealth, and it may disturb existing relations among members of the king's court, and entice attacks upon the merchants themselves for the sake of the wealth they carry…Perhaps the safest course for a king would be to drive foreign merchants away, but this action would deprive the kingdom of the wealth they bring.[39]

In our case, these small kings had hardly the power to fight against the European merchants. The foreignness therefore might have led to some uneasiness amidst the composers of these texts and their patrons which influenced the way they comprehended the Dutch (and probably all the other Europeans). Insecurities could have had

[36] This song is generally attributed to Lalon Fakir who was born in Bengal and whose songs contributed richly to the popular genre of Baul tradition. Since he did not produce any written copy of his songs and most of it were passed on orally to be transcribed later, there are contentions that some of these songs could have been productions by others that were falsely ascribed to his name. See Das, 'New Publications', p. 250.
[37] Curley, 'Tribute Exchange', *Poetry*, p. 33.
[38] Even though this seems to be a part of the *Mangal* poems, there existed indeed trading connections between Bengal and Sri Lanka with the Portuguese link. Arasaratnam, *Maritime India*, p. 156.
[39] Curley, 'Tribute Exchange', *Poetry*, pp. 4, 50.

risen from the fact that many VOC officials actually behaved like these local elites—holding villages on lease and paying regular customs to the Mughal governor.[40] This was coupled with the feeling of weakness emanating from the fact that these rulers were militarily and financially placed at a less advantageous position in comparison to the foreign merchants. But on the more relevant side, the local rulers did not really even feel the need to take the Dutch seriously or placate them, unlike the Mughals who were then a source of legitimate political power. This explains their absence in the *Mangal* poems from the seventeenth century, despite the intermittent interactions and contact with the Europeans.

But what remains evident is the fact that these locals knew the distinction among different Europeans who were nevertheless seen as a composite whole of foreigners (*smlechhas*). It was a clear departure from the time when the only white *firangis* had been the Portuguese in Bengal. Now there was a whole new variety of the Franks with different languages and lands of origin, that no doubt placed them in a distinct group that looked similar and yet did not make the locals blind to their distinctions. As Thomas Bowrey contended, there were already roughly 8,000–9,000 Christians living in the seventeenth century around the place at that moment which make us conclude that there had then been a significant European population.[41] Apparently, even if the number cannot be confirmed, it is enough to ascertain that the locals were aware of a substantial European presence amidst them.

The consciousness of the locals about the differences among the Europeans was also evident from other accounts like that of the *Bayan-i Waqi* by ʻAbd al-Karim Shahristani. ʻAbd al-Karim pointed out that there were different European settlements along the river in Calcutta and Chandannagor. Among the *firangis* who lived there, the Fransis, the Angrez, the Valendez and the Purtugez were to be found.[42] John Fryer also attested to this social diversity as being present not only amongst the Europeans but also amongst the locals themselves. He wrote that there were the Gentues, the Moguls, the Portugals, the Dutch, the English, etc. and the Parsies.[43] Even Ray in his *Annadamangal* went on to mention about the other quarters with different populations living in Bengal. He wrote how, as the Mughal general Man Singh continued to move from the European quarter to the next five quarters, he saw the Mussalmans, the Kshatriyas, the Rajputs, the Rahuts and the Bondelas living in separate spaces.[44] Segregation of spaces had certainly become prominent in the eighteenth century, but one might presume that the process must have had kicked in already in the later years of the seventeenth century. The Europeans were seen as foreigners separated in the social space of the locals and yet they could distinguish the Dutch from the French and the English from the Dutch and so on.

[40] Barangaore, Chuchura and Mirjapur were the three villages given on lease to the Dutch by the Mughals. Van Dam, *Beschryvinge*, Vol. II, Part 2, p. 24; Nationaal Archief (NA), Netherlands-The Hague(NL-Ha), Veerenigde Oost-Indische Compagnie (VOC) 1.04.02, Overgekomen brieven en papieren (OBP), inv. nr. 1421, Letter from Hendrik Adriaan van Reede in Hooghly to Governor-general Camphuys and the Council in Batavia, 16th November, 1686, f. 323r.-324v.

[41] De Graaf, *Atlas*, Vol. I, p. 207.

[42] Subrahmanyam, 'Taking Stock', p. 90.

[43] Fryer, *A New Account of East-India*, p. 189.

[44] Ray, 'Annadamangal', pp. 10–11.

In a nutshell, what can be concluded is that the locals as kings and small chiefs could identify the Dutch as one of the distinct social groups that inhabited the several quarters of Bengal. But at the same time, there was an element of foreignness in the Dutch for these locals which made it a loosely attached affair. Most of all, there was no compulsion or immediate reason for the two groups to communicate with each other on a political level for a long time, until the last decade of the seventeenth century. The Dutch were then the distant merchants for these local rulers, who saw them as useful in providing financial and naval support if required, though they were not still politically dependent upon them in Bengal's scenario. Beyond the scope of these texts, it is important to note that the Malla *rajas* of Bishnupur, known for their rise from a tribal class to a local ruling dynasty who went on to be legitimised by the Mughals as zamindars, also depicted their contacts or awareness about the Europeans through temple architecture. Scholarly contentions suggest that the figures in one of the friezes of the terracotta temple of Jor Bangla built by Raghunath Malla Dev (better known as Raghunath Singha Dev) in 1655 resembled that of a group of Europeans holding matchlocks and standing in large foreign boats (Figure 1). They seemed to be clad in short coats and trousers with headgears resembling the European hats. The possibility of them being Portuguese–Arakanese pirates could have been high, though it might be worth considering as to why they were depicted on the Brahmanic temples of the Mallas.[45] Was it because the Malla kings wanted to portray the world around them through the temple's terracotta architecture? Or was it because they wanted to show the diversity of powers that they had to contend with for their survival? Whatever the reason might have been, it does however show contact with or awareness about the Europeans, albeit within the same violence and dominance framework that was common in the expression of the local kings.

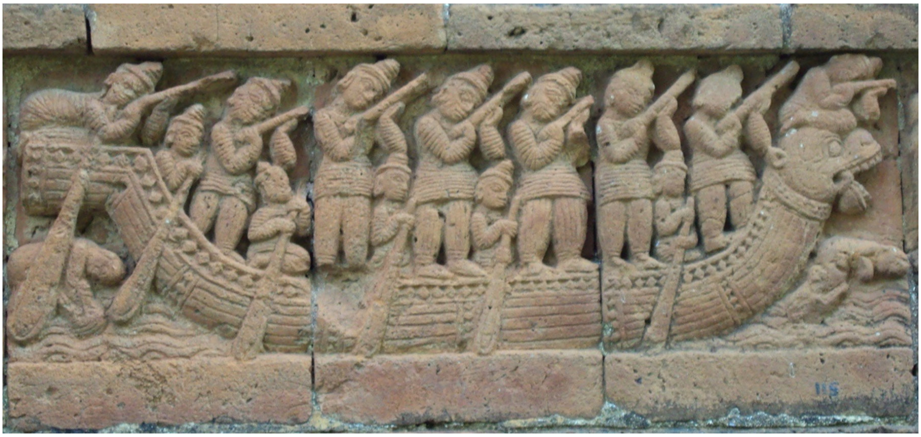

**Figure 1.** Terracotta Work on Jor Bangla Temple, Bishnupur

**Source:** Reproduced from Wikipedia.org

[45] Deloche, 'Boats and Ships', p. 12.

## The Painting

However, a painting might have different stories to tell about the perception of a very different type of local population. It might not be the best option to use a Western painting for our purpose; nor is it directly representative of the local perspective of Europeans in Bengal. But the tiny hints dropped in the painting through certain aspects that are still depicted boldly, notwithstanding all the biases, can be used for extracting some useful information.[46] Of the several paintings made on India by the Dutch, a remarkably simple yet richly informative painting happens to be that of Hendrik Schuylenburgh's depiction of 'The Dutch East India Company's Factory in Hooghly' around 1665[47] (Figure 2). Despite being a European painting, it is probably possible to extract the local perceptions and voices from it. However, before we proceed to do this, it is essential to shed some light on the context of this painting.

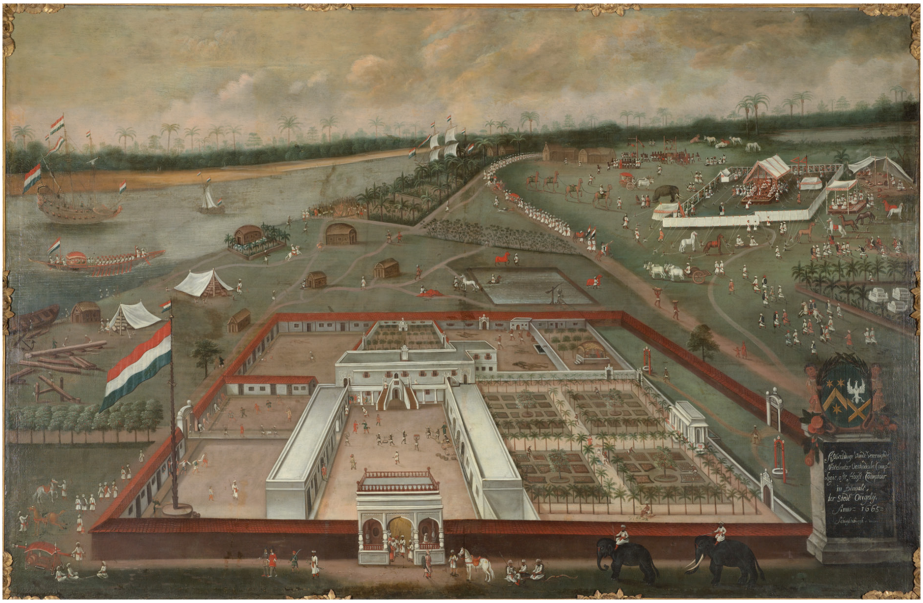

**Figure 2.** The Factory of the VOC at Hooghly in Bengal

**Source:** Hendrik van Schuylenburgh, 1665, Rijskmuseum, Amsterdam.

[46] For some studies on Dutch paintings about locals, see De Groot, 'The Earliest Witness', pp. 17–50; Taylor, 'Meditations on a Portrait', pp. 23–41; Chakravarti, 'Coasts and Interiors', pp. 95–110 and on a general overview of European paintings portraying locals, see Schmidt, *Inventing Exoticism*; Mason, *Infelicities.*

[47] Hooghly was the main factory of the VOC in Bengal. It should be mentioned that there are a couple of other paintings on the VOC factories in Bengal, possibly one of them being in Malda as shown here https://www.rijksmuseum.nl/nl/zoeken/objecten?q=bengal+schuylenburgh&p=1&ps=12&st=OBJECTS&ii=1#/SK-A-4283,1, See for more information, Gosselink, 'Schilderijen', pp. 390–409 and Chakravarti, 'Coasts and Interiors', pp. 95–110.

Schuylenburgh (also spelt as Schuylenburgh sometimes), its creator, was born in Middelburg in c. 1610 and his name appeared for the first time in the St. Lucas guilder-book for the year 1644. After a brief pause, his name reappeared as an administrator for the years 1653, 1655–60, and 1668.[48] Martine Gosselink who has worked extensively on this painting has pointed out that Schuylenburgh could have had possibly made this painting during his stay in Asia between the years 1661–68 while being enrolled in the service of the VOC.[49] She further argues that the painter did not capture the happenings or incidents on spot but rather portrayed a concoction of everything he had seen there, including the director of Bengal's ceremonial journey from the factory at Hooghly to that of Kasimbazaar[50] (Figure 2.1). This can put aside for the moment all debates and contentions about the painter relying on hearsay, rather than being himself present in Bengal. There are further details in this painting that might be worth discussing but to save time and space, I would stick to the scope of our subject—the local perception.

The first thing that needs to be kept in mind is the fact that this was a painting meant for the European audience and made at the behest of the VOC, possibly the Zeeland chamber.[51] The striking contrast between the scene inside the factory with the scene outside its walls thus appear to be much more comprehensible in this light

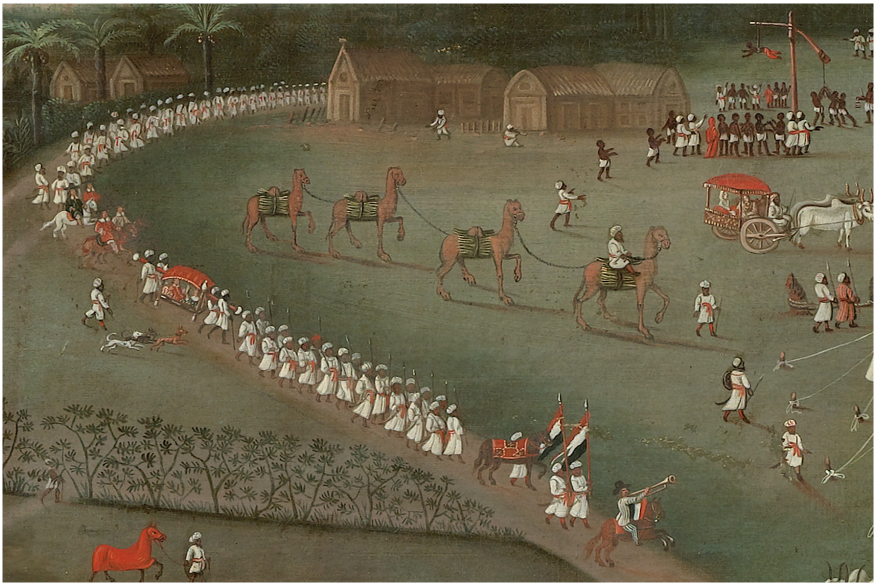

**Figure 2.1.** A Zoomed-in View of the Probable Ceremonial Journey of the Director of Bengal from Hooghly to Kasimbazaar

**Source:** Hendrik van Schuylenburg, 1665, Rijksmuseum, Amsterdam.

[48] See for more biographical details on the webpage of the *Rijksbureau voor Kunsthistorische Documentatie* https://rkd.nl/en/

[49] Gosselink, 'Schilderijen', pp. 397–98.

[50] Ibid., pp. 398–401.

[51] Ibid., p. 398.

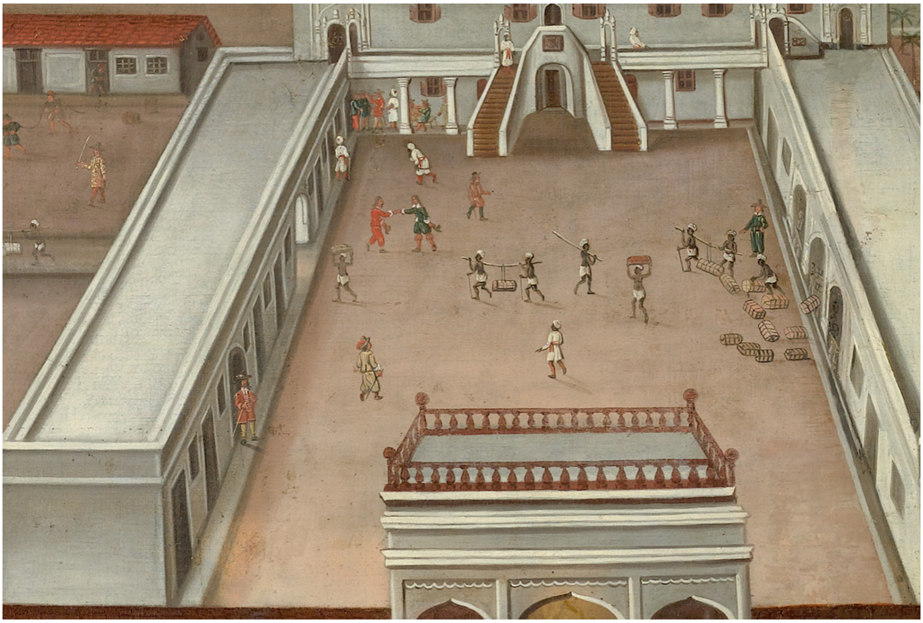

**Figure 2.2.** Zoomed-in View of the Scene Inside the Factory
**Source:** Hendrik van Schuylenburg, 1665, Rijksmuseum, Amsterdam.

of explanation.[52] Schuylenburgh attempts to show the neatness and order inside the factory with the people going around doing their usual business on time (Figure 2.2). This is in stark contrast to the picture beyond the factory's walls where there is utter chaos and confusion (Figure 2.3). The local people are shown to be walking around aimlessly and enjoying music or conversations without working unlike the Company's ambience inside its walls. Besides this, the depiction of *Sati* or widow immolation practice (Figure 2.8), the festival of *Gajan* that is referred to in the Dutch accounts as hook-swinging (Figure 2.10), and the display of music with fun and frolic (Figure 2.9) appear to be loosely placed as though it was a common everyday scene for Bengal's social setting.

Festivals, arguably, mark days of subversions where daily social norms and moralities are broken in the spirit of fun and freedom. On the day of Holi (festival of colours), for example, subtle sexual remarks and alcohol consumption is encouraged that would otherwise not be accepted in normal situations. This shows how festive occasions allow the subversion of certain social rules that dominate daily social interactions. Schuylenburgh's portrayal of these elements in a painting of the factory in Hooghly with its local surroundings then seem to be a forced depiction of subversions as normal features. It is a festive image imposed unreasonably on a regular day, making it look like a normal, everyday scene from the local lives. Notwithstanding the fact that such exaggerations were meant to serve as excellent food for the exotic hunger of European

[52] For an analysis of the local landscape in this and other European paintings, see Chakravarti, 'Coasts and Interiors', pp. 104–06.

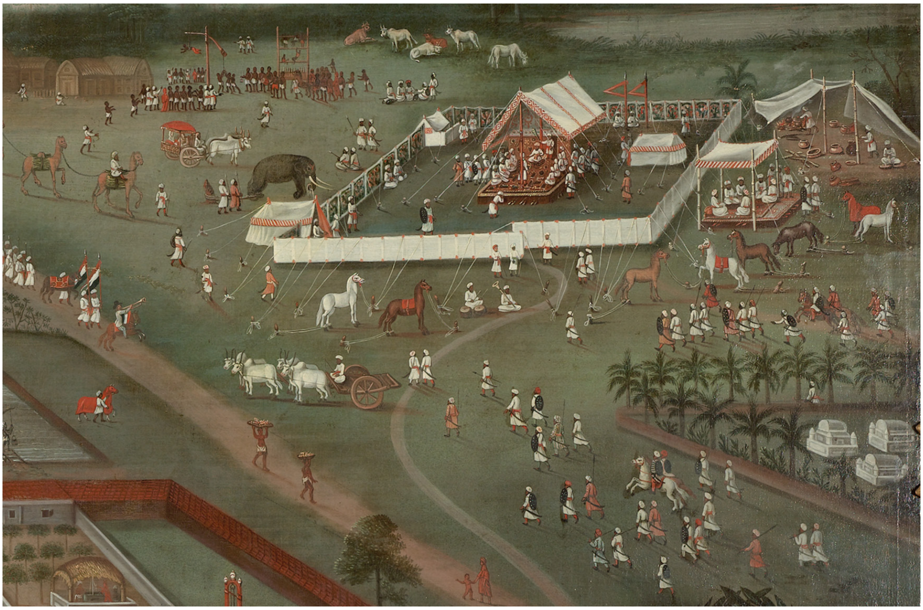

**Figure 2.3.** Zoomed-in View of the Scene Outside the Factory

**Source:** Hendrik van Schuylenburg, 1665, Rijksmuseum, Amsterdam.

elites, the painter does not, however, make an effort to hide certain Dutch–local interactions. One spots them standing together and talking with the locals freely. Given that the *Statutes of Batavia* where the rules of the Company servants' behaviour were codified, forbade explicitly on the mixing of Company servants with the locals, the painter's making no qualms to hide these local interactions does say something about the situation in Bengal.[53] It provides us with a very different overview from that of the local texts where the prominent feelings spelled out were distance and discomfort.

To begin with a basic aspect that would constitute the obvious element of encounters, one can speculate on the local perceptions of the appearance of the Dutch Company and its servants. It is important to point out that the locals here indicate those seen in this painting, working inside the factory—mostly simple villagers or small brokers and merchants. These were no kings, politicians or wealthy traders with crucial social status. These locals comprised a part of the population of the village of Chinsurah (in Hooghly) which happened to be one of the three villages that had been given to the Company in lease by the Mughal subahdar, Shah Shuja.[54] There is hardly any source for the time that has preserved their thoughts and opinions. But even though it is hard to find any textual evidence for knowing their observations on the Dutch, we can safely ascertain that seeing foreign traders, travellers and diverse ethnicities was not something new for these locals. They have had experiences of dealing with different people from different parts of the globe for centuries already, making it plausible that they

[53] Van der Chijs, *Nederlandsch-Indisch Plakaatboek*, Vol. I, pp. 124–25; Vol. II, p. 548.
[54] NA, NL-Ha, VOC OBP, inv.nr. 1421, Letter from Van Reede to the *Heren XVII* dated 9th December, 1686: f. 75v.

did take notice of the Dutch presence but did not find it something alarmingly new or unusual. In 1636, when the VOC set up its first settlement near Hooghly, it started out as a dwelling of straw-mats and bamboos accompanied by a mud warehouse in the midst of the common houses.[55] By the middle of the century, the factory had expanded and become more solid. But as it can be seen in the painting made in 1665, there still remained a substantial amount of openness and contact with the world beyond the walls.[56] It was only later in that century that greater investments were made in closing the factory with higher walls and making it fortified, thus cutting off the merging of the local and the Dutch spaces.[57]

What did this imply for the local people? It is not surprising to assume that in the early years the Company would not have had evoked any amount of respect or fear in their minds. Not that they would have not noticed the VOC's arrival but that it would not have had been particularly impressive. However, there must have been tremendous changes in their perceptions towards the end of the century when the Company already built a solid factory with well-equipped warehouses and gardens, separated by high walls from the outer chaos. The image of the company must have had then been elevated to a significant extent so as to leave an imprint in the minds of the locals. While this was about the Company as an institution, there were still the Company's individual officials. They interacted with these locals on a much closer and personal level, sometimes even a bit too much against the wishes of the directors in the Dutch Republic.

The desire to segregate the Dutch from the non-Dutch was an ideal situation that the VOC wanted, which is partially reflected in this painting. But it did not always happen. As is evident here, the involvement of a large number of locals as manual labourers in the factory meant that they helped the Dutch men struggling to learn the ways of Bengal and its people for fitting into the picture. The locals on the other hand, were financially dependent on these Company personnel, and were compelled to adapt to their needs and lifestyle. They responded to the Company officials' demands by offering them

[55] Van Dam, *Beschrijvinge*, Vol. II, Part 2, p. 2. The first VOC presence was in Pipli in the year 1629 followed by Ballasore, Hariharpur and finally Hooghly when their forerunners in Bengal, the Portuguese were ousted from their strongholds in the ports of Hooghly and Chittagong by the VOC. See Van Dam, *Beschryvinge*, Vol. II, Part 2, p. 14; Gommans et al., *Grote Atlas*, pp. 28–29, 383. The administration of the Company in Bengal was clubbed together initially with the administration of Coromandel, but the trade in commodities like silk, sugar, textiles, rice, cloth, saltpetre and such other grew so fast and became such increasingly profitable in Bengal that a separate trade account had to be opened in 1655 with a new special post for the director of Bengal. Unfortunately, a flood in Hooghly in the year 1656 caused a massive damage to the Company's lodge leading to it being temporarily shifted to Kasimbazaar. See Van Dam, *Beschrijvinge*, Vol. II, Part 2, p. 17 and Gommans et al., *Grote Atlas*, p. 399. For more on the VOC in Chinsurah visit http://dutchinchinsurah.com/timelines.php

[56] We know that this is a true depiction because of the Van Reede Committee reports later that suggested changes and there we can see the exact layout of the factory plans. See Gosselink, 'Schilderijen', pp. 399–400; Gommans et al., *Voor-Indië, Perzië, Arabisch Schiereiland*, p. 399. Schouten in 1663 also writes about the factory in Hooghly as being '*onbemuurd en open*' (unwalled and open) so that the entire factory could not be separated very well from its surrounding villages and the inhabitants. Schouten, *De Oost-Indische voyagie*, Vol. III, p. 373.

[57] NA, NL-Ha, Instructions and Regulations, made by H. A. van Reede tot Drakestein, lord of Mijdrecht, as the commissioner appointed by the *Heren XVII* for the director and Council in Bengal Copie 1687, Hoge Regering Batavia (HR), inv. nr. 241, folio not numbered.

a plethora of services. These relations were given a formal façade though it remained mostly informal and developed into complicated, hierarchical bonds beyond the *Heren XVII*'s (board of directors in the Dutch Republic) reach.[58]

In this painting one can see gardeners, stable workers, coolis, water bearers and other local people working for the factory (Figures 2.4, 2.5 and 2.6). This can be corroborated by a list drawn up by Hendrik Adriaan van Reede for showing the kind of services used in the factory of Hooghly. The list ranged from scribes, brokers, translators, porters, peons, market-goers (for helping with groceries) to water-bearers, washer-men, barbers, cooks for the hospital, rope-makers, carpenters and so on.[59] The officials also enjoyed personal slaves.[60] It leads us to contend that the Company and its officials were seen as providers of opportunities for work and economic gain by these ordinary locals who welcomed the Dutch presence in Bengal. Besides this, the finesse that some of the VOC officials developed in imitating the local elites and putting on fine clothing meant that it put them in the eyes of these ordinary men to a privileged social rank. It was proof that they had money to spare which made the small brokers and wage labourers perceive the Dutch as harbingers of economic opportunities, and powerful elites.

This painting provides more of an ideal depiction of what the administration in the Republic would have had wanted its officials to wear, that is Western clothing. But there is another reality that is recorded in the Company's rule-book. The regulations there hark repeatedly about the importance of adhering to European clothes and not trying to imitate the local elites and their ways of attire, indicating that the VOC officials were precisely doing otherwise.[61] Indeed, this conjures up an image of the Dutch in

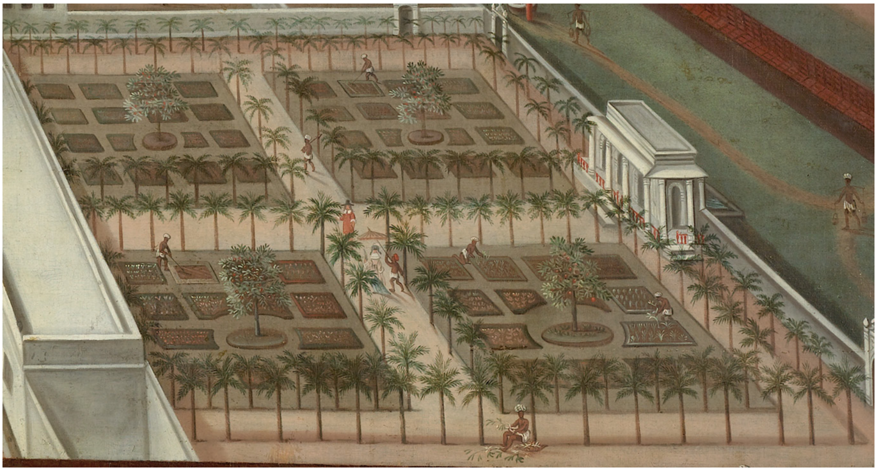

**Figure 2.4.** Zoomed-in View of the Factory's Garden with Locals as Workers (Gardeners, Water-carriers, etc.)

**Source:** Hendrik van Schuylenburg, 1665, Rijksmuseum, Amsterdam.

[58] For more on the Company officials' political motives see Antunes, 'Introduction', pp. 13–20.

[59] NA, HR 241, folio not numbered. On the rope-making practices in Bengal, see De Graaff, *Oost-Indise Spiegel*, p. 84.

[60] NA, HR 241, folio not numbered.

[61] Van der Chijs, *Nederlandsch-Indisch Plakaatboek*, Vol. I, pp. 470–71, 595–96; Vol. II, pp. 119, 306.

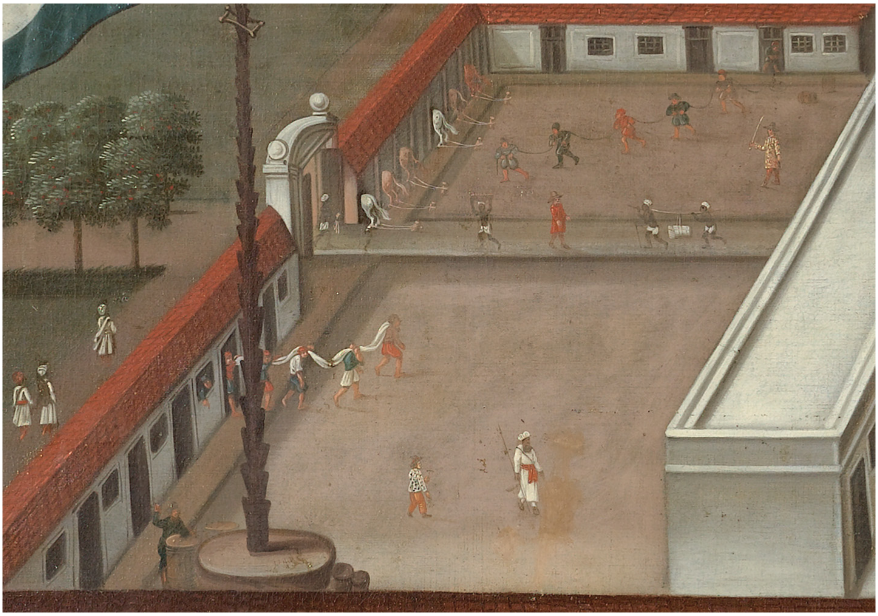

**Figure 2.5.** Zoomed-in View of the Locals Working as Coolies, Porters, Stable-workers, etc.
**Source:** Hendrik van Schuylenburg, 1665, Rijksmuseum, Amsterdam.

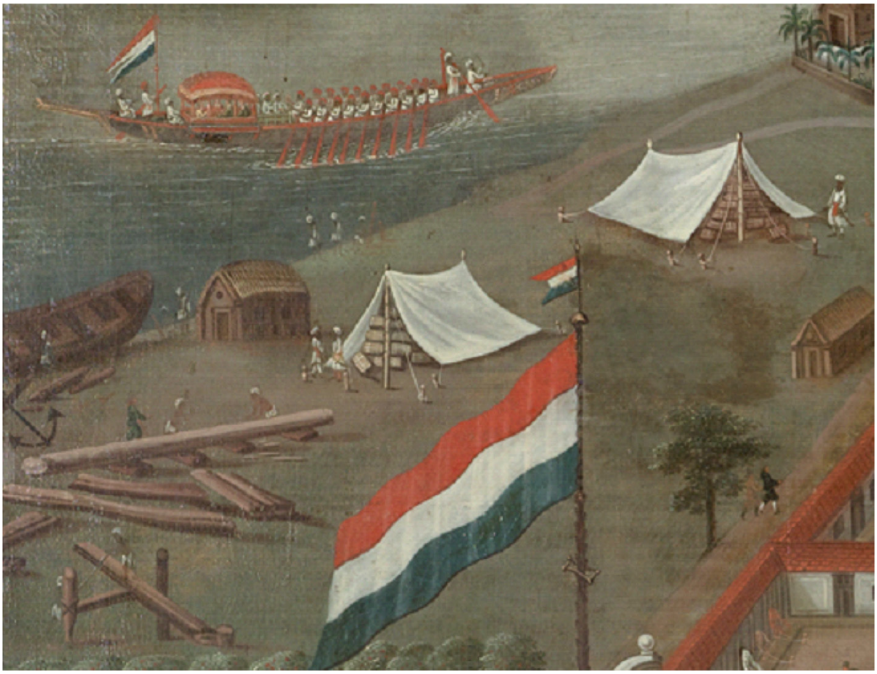

**Figure 2.6.** Zoomed-in View of the Locals Working as Carpenters, Rowers, Ship-builders, etc.
**Source:** Hendrik van Schuylenburg, 1665, Rijksmuseum, Amsterdam.

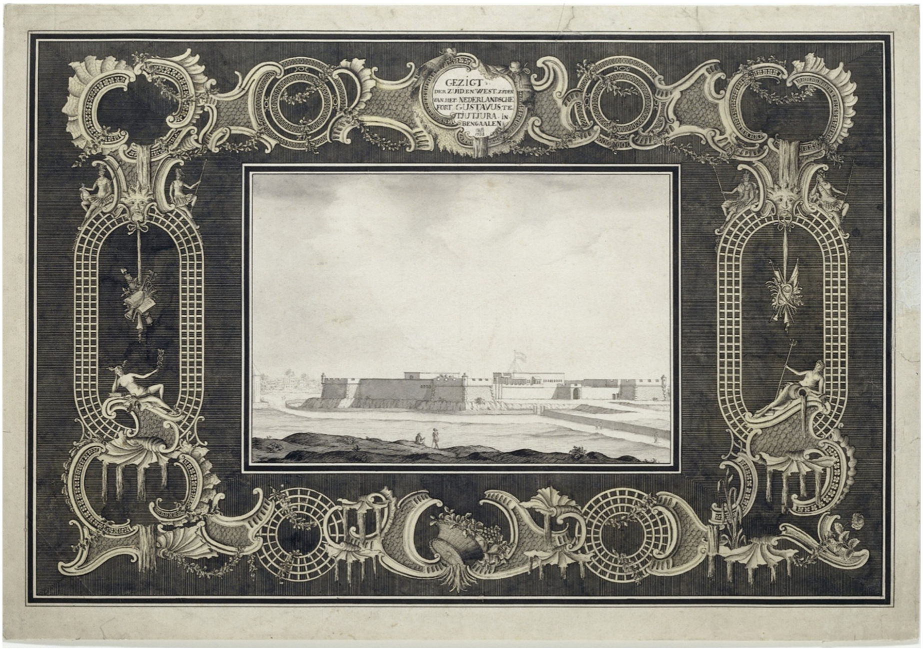

**Figure 3.** The View of the Company's Fort Gustavus at Chinsurah in Bengal in 1770

**Source:** Johannes Rach, Rijksmuseum, Amsterdam.

the local eyes that seems to be very different from the observation of the Bengali texts. Here the local perception, of the Dutch, seemed to be that of rich magnates who had to the power to be able to command their obedience. What differs from the textual evidences in terms of the portrayal of the dynamics of local–Dutch relations is that Schuylenburgh renders no inkling of friction in his painting. The absence of arms or fortifications in the factory is particularly striking and the Dutch official reports also do not mention about any ammunitions in the factory.

This is very much unlike the last decades of the seventeenth century when the Dutch started erecting a well-equipped Fort Gustavus at the same place[62] (Figure 3). The fact that Schuylenburg makes no effort in showing streaks of fear or tension in his painting, says a lot about the ease with which the Dutch interacted with these ordinary locals. The painting, in fact, exudes the comfort of a relaxed and routine summer day with marks of languor at some places while others went about doing their usual work. When Hendrik Adriaan van Reede came to investigate the factory in 1685, he complained in his reports to the *Heren XVII* (board of directors in the Dutch Republic) about the noise

[62] The construction of Fort Gustavus was completed by Jan Sichterman in the eighteenth century. Van Diggelen and Brood, *Jan Albert Sichterman*. There were orders issued by the *Heren XVII* on 23rd June, 1700 for strengthening further the lodge and factory in Hoogly. See NA, NL-Ha, VOC, Zakenindex op de uitgaande missiven van de kamer Amsterdam aan de kantoren in Indië. Met bijlagen, inv. nr. 345., folio not numbered.

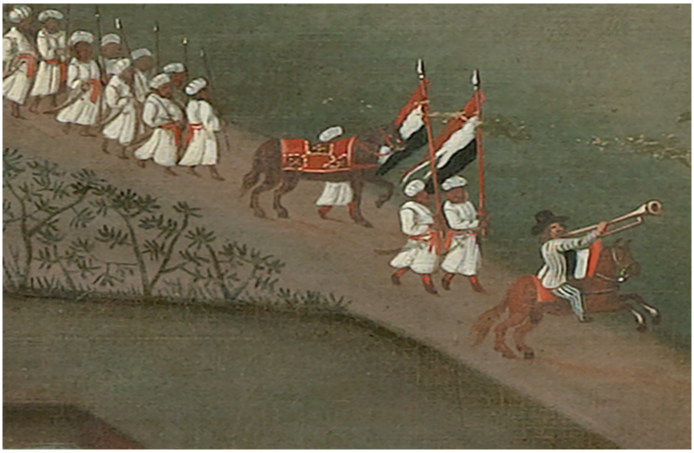

**Figure 2.7.** Trumpets with the Ceremonial Journey of the Director of Bengal (Zoomed-in Views)
**Source:** Hendrik van Schuylenburg, 1665, Rijksmuseum, Amsterdam.

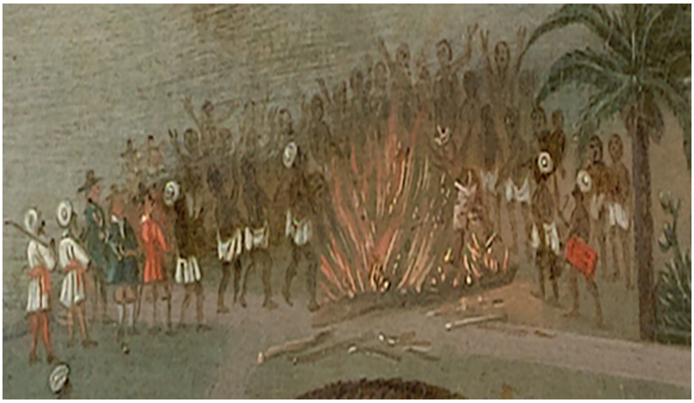

**Figure 2.8.** *Dhaak* with the Performance of Sati (Zoomed-in Views)
**Source:** Hendrik van Schuylenburg, 1665, Rijksmuseum, Amsterdam.

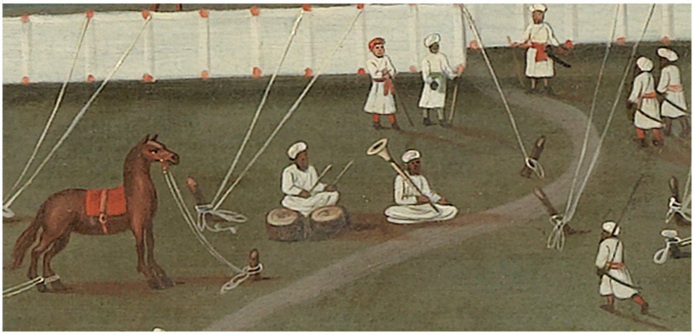

**Figure 2.9.** Musical Accompaniment of *Shehnai* and *Tabla* at the Local Ruler's Camp (Zoomed-in Views)
**Source:** Hendrik van Schuylenburg, 1665, Rijksmuseum, Amsterdam.

of this place that could go out of hand sometimes.[63] Through this painting, one can almost hear the din and hustle-bustle—the trumpet, the *dhaak*, the musicians with the *shehnai* and the *tabla* and the voices of the people everywhere chatting and laughing and walking by (Figures 2.7, 2.8 and 2.9). However, given that the VOC reports were almost always filled with references to local sounds and music as disturbing noises for the Dutch settlements in other places, one might be inclined to assert that this was more of an exaggerated depiction of Bengal rather than an honest one.[64] The more chaotic the situation could be depicted as, the higher the moral and legal claim it gave to the Dutch officials. In some way,the audience of this painting is almost led to believe that the Dutch had greater acceptability among these ordinary locals for the order that they had brought in Bengal. This definitely betrayed their motives of gaining legal control over the locals, by discounting their order and law situation. However, if we turn the tables around, it is possible to conclude that from the perspective of these locals, the Dutch were indeed seen as powerful elites who had the money and the might and should therefore be seriously reckoned with.

Despite the multitude of languages that were spoken in Bengal at that time, it was possible for the locals to distinguish the Dutch from the other foreign merchants. References to brokers using Dutch becomes clear only from the eighteenth century sources but for the seventeenth century, it was mostly Portuguese Creole and sometimes Bengali, Hindustani and Gujarati languages.[65] For the inhabitants of Bengal who worked with the VOC, it could not have been much of a problem then since the Company officials themselves also adapted to the local languages and customs to a large extent. It is not uncommon to find instances where Indian brokers supported certain VOC officials, despite the latter's being charged of illegal trade, on grounds of proximity with them. The complementary reason was that these officials could speak Indian languages and thereby had developed better connections with their surroundings and the local inhabitants.[66]

This painting conveys a far less troubled perception of the locals about the Dutch. They seem to be sharing moments in history and participating in each other's culture, habits and lifestyles unlike the distance reflected in the textual sources. However, if we study this painting to dig out the local voices representing the brokers, we can conclude that they still perceived the Dutch as their employers and were absorbed subtly into the Company's administrative framework. As the instructions from the *Heren XVII* emphasised more and more on the duties of the VOC officials as Christian merchants who should protect themselves from the corrupting influence of the Indians, the practices seemed to reflect huge discrepancies and a further drifting away from the official

[63] NA, HR 241, folio not numbered.

[64] De Groot, 'The Earliest Eyewitness', p. 40; Van der Chijs, *Nederlandsch-Indisch Plakaatboek*, Vol. II, p. 227; Subrahmanyam, *Forcing the Doors*, p. 13.

[65] Languages used can be understood from the local languages mentioned in the archives from which they are translated to Dutch. See NA, VOC, 1422, About the accounts of the Company's factories in Bengal during the time of Jan Pit in 1683 from the month of August, f. 1085v.-1086r., f. 1139r., f. 1140r.; NA, NL-Ha, VOC, Ingekomen stukken van de Raad van Justitie in Batavia bij de Heren XVII en de kamer Zeeland, Files concerning the judicial process against Nicolaas van Schagen, ex-director of Bengal, guilty of indulging in illegal private trade, inv. nr. 9521, folio not numbered.

[66] Gaastra, 'Constantijn Ranst', p. 130.

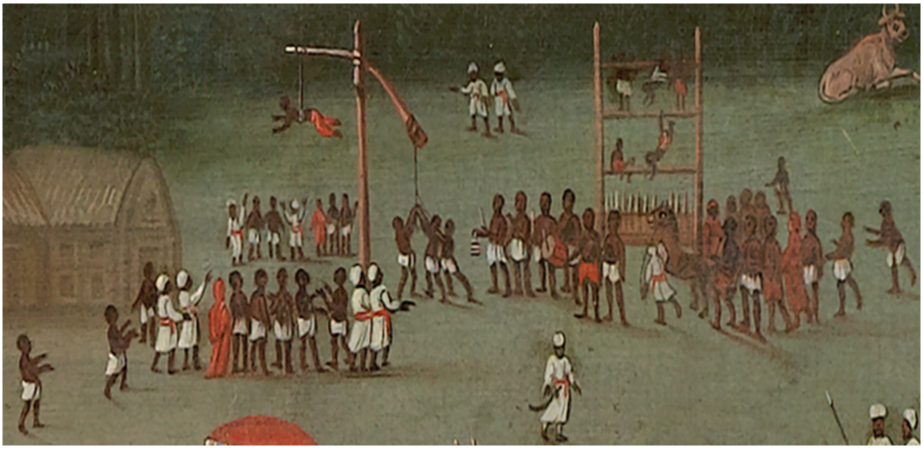

**Figure 2.10.** The Depiction of the Festival of *Gajan* or *Charak* Fair Referred to as 'Hook-swinging' in Dutch Sources

**Source:** Hendrik van Schuylenburg, 1665, Rijksmuseum, Amsterdam.

injunctions.[67] This very painting shows the Company servants standing at ease amongst the regular village houses, mingling freely with the locals. It also shows some of them watching the fire ceremony or riding towards the local king's camp where the festival of *Gajan* is going on[68] (Figure 2.10). There also seems to be Indian soldiers from the royal camp entering and leaving the factory at ease (see Figures 2.2 and 2.3). Despite rules forbidding such closeness, the painter's free depictions of such local–Dutch mixing almost compels us to believe that these two groups had a greater symbiotic relation.

This contention can be supported by Wouter Schouten's accounts. During his period of service to the VOC as a surgeon, he wrote about his stay in Bengal. There he narrated about some Company personnel being used to visiting the houses of wealthy Indian merchants and being treated with betel and areca nuts.[69] Nicholaas de Graaff, another surgeon serving the VOC, wrote similar stories about Bengal when his ship reached its shores in 1668. He mentioned how the captain (*schipper*) of the ship along with the others, packed their goods and went inland, to rent a lodge for staying there as long as the ship lay anchored. It was at this time that De Graaff saw the Dutch officials go around with their Bengali friends and other *bania* (commonly used European term for referring to non-Islamic local merchants in India) merchants every day to conduct illegal trade in that region.[70] Even though we accept that they all lived in different quarters,

[67] One of the sources say, '… but if we follow the same ill-manners and defects of the inhabitants, we would burden ourselves to the extent of making us incapable of several things, and that would be the cause not only of contempt but also of our incapability in serving our masters properly'. NA, HR 241, folio unnumbered. This stereotyping even extended to the legal domain where the involvement of locals in judicial procedures was questioned. Van Reede himself wrote that it was believed that guilty company officials knew the art of hiding their own secrets while the untrustworthy nature of 'the heathens and the moors' rendered them useless in acting as witnesses against the officials. See NA, VOC, OBP 1421, f. 19v.-20r.

[68] See for details on *Gajan* Nicholas and Curley, *Rites of Spring*.

[69] Schouten, *Oost-Indische Voyagie*, Vol. III, p. 371.

[70] De Graaff, *Oost-Indise spiegel*, p. 93.

there seemed to be no restrictions in letting everyone move freely across all these quarters. For the ordinary local inhabitants, it was all the more a favourable situation since they could work for anyone and make money irrespective of group distinctions.

We can get a glimpse of this further when Schouten writes about their ship getting stuck in a sandbank in Sankrail (50 km away). Here they had to wait for the tide to return before their ship could go out to sail again. For that afternoon, he gives an account of how the entire sandbank was transformed into a marketplace with the local inhabitants selling fresh food and other goods to them.[71] Similarly, he also talks about two boats of Bengali men who offered to work on the Dutch ship for wages when they needed protection against the Arakanese pirates on their way to Hooghly on the Ganges.[72] On his arrival in Hooghly, Schouten wrote about the almost institutionalised provision of lodging, food and personal attendants for the Europeans. He wrote:

> We hired, as per the Bengali custom, a house to live in for a few days as if it was our own and put in all our luggage there. Each of us hired a Muslim servant who looked after us and provided us with everything that we needed. Our landlord was a black Mestizo … In Hooghly he possessed a few Bengali style houses where he employed various black male and female slaves who not only served him but also helped him in earning his bread. We arranged for meals to be served to us every morning, afternoon and evening with this Mestizo who happened to be both our landlord and neighbour now. The food prepared and served to us by his slaves (both men and women) were so good that we had hardly anything to complain against.[73]

If this account provides a peek into the booming slave trade around the Bay of Bengal at that time, it also shows how the opportunities from this trade were exploited by all elites—both the locals and the Europeans. It should also not be forgotten that the Company did not have just its higher officials but of course its ordinary sailors and workers. These men came overseas from Europe on temporary contracts but were fast enough to study the local setting and learn the slips and gaps of the system.[74] The official reports are full of complaints about European contract labourers deserting the VOC by breaking their contracts and fleeing to the English and other European factories for better wages.[75] If caught, they faced punishment but if not, there was nothing that the Company could do to enforce their monopolistic rules in such a competitive and pluralistic space. These workers of the Company also formed a reliable group of customers who bought alcohol, prostitutes and slaves from the local villages.[76]

[71] Schouten, Vol. III, p. 368.
[72] Ibid., pp. 366–67.
[73] Ibid., p. 371.
[74] It should be noted that working for the VOC was the least desired labour sector among the Dutch in the seventeenth century even though the Company remained one of the biggest employers for labourers. Many of them were even criminals escaping punishment by working for the Company and thus being away from the Republic.

Prak, 'Loopbaan en carrière', p. 135; Wagenaar, 'Extortion and Abuse', p. 737.

[75] NA, NL-Ha, Collectie Hudde (CH) 1.10.48, inv. nr. 36, folio not numbered; NA, VOC 1421, f.79r., f.92r.v.; NA, NL-Ha, VOC, OBP, Report from H.A. van Reede to the *Heren XVII* in Middelburg, December 1687, inv. nr. 1435, f. 16v.-17v.; Van der Chijs, *Nederlandsch-Indisch Plakaatboek*, Vol. I, pp. 92, 125, 214, 248, 591; Vol. II, pp. 105, 170–71.
[76] Schouten, *Oost-Indische Voyagie*, Vol. III, p. 372.

The local perception of the Company, as can be deduced from this painting, was thus that of potential employers and powerful elites. The locals here obviously belonged to the socially lower ranks who were bound to the Company for their financial gains. Since the base for sustaining their relation was economic means, they had little authority to cause hindrance to the Dutch. Their relation could be seen as symbiotic, though certainly not freed from hierarchical implications. Unlike the local rulers of Bengal, these people worked with the VOC more frequently and daily and therefore had a much higher level of interaction. The smaller merchants or brokers experienced no visible and imminent threat from the Company or its officials but nobody can deny the fact that conflicts never occurred between them and the officials. Such conflicts between the officials and the brokers were however as common as between those same brokers and the Mughal merchant-officials.[77] As regards the ordinary villagers, they often got unwittingly absorbed into the Company's administrative domain, triggering off a slow but intense process of political contestation of the Company officials against the Mughal nobles. These group of locals with a subordinate social position, had therefore more uneven perceptions of the Dutch owing to their closer contact and financial involvement, than the *Mangalkavya* composers and their patrons as discussed in the previous section.

## A Legal Case

The last source pitches theoretical and visual evidences against the practical outplay of power relations in the politico-juridical sphere. How did the locals perceive the Company and its officials when it came to issues of exercising their authority and jurisdiction? Through the study of a legal case recorded in the VOC files as 'The Death of Boloram's Widow' (written in the sources as Bolleram) around 1672–73, the answer to this question is unfurled which helps in turn, to add an extra layer to the local–Dutch dynamics in Bengal.[78] Both Om Prakash and F. S. Gaastra have written about this incident before but the local perspective of the story in both cases has been too quickly gleaned over.[79] But before proceeding with this task of revisiting this case for the missing conclusions, it is necessary to understand that the VOC in Bengal had no territorial power and no separate governor. The Company did not possess any judicial rights over the inhabitants of Bengal, nor did it have any control over the locals whose services were used by them. The Company here, with its director and council was in fact subjected to the authority of the Mughal administration and its laws in Bengal. Most of its civil cases, involving its officials were delegated to the *Raad van Justitie* (Council of Justice) in Batavia which served as the highest judicial court for the VOC overseas. Under such circumstances, this case shows how a combat-zone was created for the Mughal officials in Bengal struggling to assert their power and authority against

[77] Das Gupta, 'The Maritime Merchant', p. 100.

[78] NL, NA-Ha, VOC, OBP, Stukken van Constantijn Ranst 1675, inv. nr. 1306, f. 205r.-211r.; NA, CH 36, folios not numbered.

[79] Prakash, 'The Dutch East India Company', pp. 286–87 (Om Prakash reads the name as Bhola Ram and later Gaastra too uses the same form, though I choose to use Boloram which sounds phonetically more plausible in this case); Gaastra, 'Constantijn Ranst', pp. 126–36. See also Van Meersbergen, 'Kijken en bekeken', pp. 201–16.

the Company officials. The locals in this case were not ordinary villagers or small rulers but important political figures holding key administrative positions. Their perceptions of the Dutch were therefore quite different and it is precisely this dimension that has been examined here.

It all began when Boloram, the Company's senior weaver (*masterhaspelaar*) died without clearing his debts to the then *faujdar* of Hooghly, Malik Qasim.[80] Qasim desperate to get his money back attacked the VOC director, Constantijn Ranst as Boloram's employer for paying back the amount. A dishevelled Ranst, not knowing what to do, refused to pay back the sum. As pressure from the Mughal authorities mounted, Ranst went to Boloram's place for clarification. On failing to find any proper account of the debt and any property to use for clearing it, Ranst took hostage of Boloram's widow and her son. They were brought to the Company's lodge and interrogated for a long time before being released later that day. The next morning was all about the news of the widow's death which spread in the neighbouring villages rapidly as Malik Qasim held her corpse and showed it around. Along with the then *subahdar* of Bengal, Shaista Khan; Qasim blamed the Company for this death and withdrew permission to let them trade until and unless the debts were repaid.[81]

This jeopardised the VOC's very existence in Bengal and a panicked Ranst had to report back to the directors in the Republic. The *Heren XVII* finally agreed to give the money required but the matter went out of hand when charges of corruption were simultaneously slapped against Ranst besides his involvement in this incident.[82] He was put on trial at the *Raad van Justitie* and at the same time, a diplomatic mission to the Mughal emperor, Aurangzeb was sent for negotiating on this payment. Since it involved Mughal officials, this case was put under the jurisdiction of the local Qazi who too tried Ranst and the Company, and gave his judgment.[83] The accusations of Malik Qasim and Shaista Khan were supported by two other Company officials called Jacob Verburgh and Herman Fentzel who alleged Ranst of torturing the widow to death. Verburgh was supported by the *diwan* (chief revenue officer) Rai Nandalal as well as other administrative elites who had been allies of the *diwan*.[84] The fact that the Company had to pay the demanded sum in the end shows that the *faujdar* and his group must have had had the upper hand at the Qazi's trial.

[80] See for a basic overview on the functions of the Mughal *faujdar* Edwardes and Garrett, *Mughal Rule in India*, pp. 184–85. The VOC often refers to a *faujdar* as the '*gouverneur*' or governor of a certain place.

[81] A Mughal *subahdar* was one who was put in charge of a certain *subah* or province under Mughal rule. So for *subah* Bengal, a Mughal official as the governor of that *subah* would be appointed and he would be called the *subahdar* of Bengal. These were however temporary tenures and the *subahdar* of a particular *subah* could be changed from time to time. See for more on this Chatterjee, *Merchants, Politics and Society*, p. 33. The VOC sources usually referred to the *subahdar* of Bengal as the *Nawab* of the Dacca or the nawab of the political capital of Bengal at a given point of time. For more details on the Mughal administrative positions, see Singh, *Town, Market.*

[82] After Ranst's assistant, Hofmeester died his wife gave away his articles to another Company official, Van Leenen who used these articles with the anti-Ranst faction to accuse Ranst of corruption.

Gaastra, 'Constantijn Ranst', pp. 130–31.

[83] The document on the Mughal side of this story has not been tracked down and therefore cannot elaborate at length the details of those proceedings. It might be worth noting that in some instances, even the *faujdar* could hold his court with the qazi for relevant matters. Siddiqui, 'The *Faujdar* and the *Faujdari*', pp. 246–48.

[84] Gaastra, 'Constantijn Ranst', p. 130.

This case says a lot about the relations and the way the Company was seen through the local lens. First, it should be noted that the locals we speak of here are of a different order and power—they are the big 'portfolio-capitalists' who clearly had the power to manipulate the Company officials.[85] They consisted of the likes of Shaista Khan who combined politics and commerce to strengthen their positions. It was obvious that they did welcome the presence of foreign merchants as long as they did not strive to achieve a similar profile as theirs on the Indian waters.[86] The Company officials were trying to grab their commercial shares as well as encroach into their administrative space. It was therefore not comfortable for them to watch the growth of the VOC in Bengal, both military and financially, since that meant making the lower political actors (mainly the zamindars and local kings) in the subcontinent dependent on European assistance.[87] These Mughal administrator-cum-merchants had financial stakes and did not restrain from using their political powers to materialise their desires. Allowing the VOC to flourish would have meant sharing profits and power with more people than they would have had preferred. Thus, the sight of the VOC officials in this case would not have been pleasing to these locals.

Constant attempts of the VOC officials to challenge the Mughal authority, resulted in tension and hostility, that underlined this relation. The Company survived on the basis of different *firmans* (imperial orders) granted to it at different points of time with permissions to have free trading rights and issue *dastaks* (trading licences) to the merchants working under them.[88] But the Mughal officials reported misuse of these *dastaks* by the Company officials in Bengal. The VOC was accused of giving away *dastaks* to merchants who had no business with the Company at all or allowed to be used by local smaller merchants for their illegal profit.[89] This led to continued distrust on both sides, while at the same time they were compelled to work together. Mere coexistence, thus, with an air of detachment was not possible because these Mughal officials had to interact with the Company servants and regulate their activities everywhere. Unlike the smaller rulers and intermediate landlords who could have had afforded to maintain occasional contacts with the Dutch, the Mughal officials were bound to mingle with them regularly as part of their administrative duties.

Being the bigger fries with huge political powers, they were therefore not subjugated by the VOC but hovered above them and tried to bend things to their advantage through legal orders. It was therefore a relation where the locals in terms of these Mughal officials saw themselves as the legitimate powerholders who were to direct the VOC's trading activities to their commercial benefit. This was possible since the

[85] Subrahmanyam and Bayly, 'Portfolio Capitalists', pp. 401–24.

[86] For more on these administrator-cum-merchants, see Chandra, *Essays in Medieval Indian*, pp. 227–34; Sarkar, *Life of Mir Jumla*, pp. 216–18; Flores, 'The Sea and the World of the Mutasaddi', pp. 55–71; Mukherjee, *Strange Riches*, pp. 13–15, 187–88.

[87] Ranst himself wrote to the VOC why Shaista Khan complained against him and the Company saying that it hampered his own profit and enterprises. NA, CH 36, Letter from Constantijn Ranst in Amsterdam to the *Heren XVII*, 12 May 1682, folio not numbered.

[88] Prakash, 'The Dutch East India Company', p. 279.

[89] Ibid., p. 279; NA, NL-Ha, VOC, OBP, inv. nr. 1422, Translated missive written in Persian by Nawab Shaista Khan (spelt as Chahastachan) to Hendrik Adriaan van Reede on 6th June, 1686 in Bengal, f.1255r.-1256r.

Dutch in Bengal had the financial resources to threaten these local officials but lacked the required legitimate authority to counter them in the political space. Far from a symbiotic relation, this was therefore a hierarchical equation where local Mughal *subahdars* treated the Dutch as their subjects who could be tried and accused, if they did not take into recognition the local laws.[90]

Even though things seemed to be apparently troublesome between the Mughal and the VOC administration at an institutional level, it can be argued that most of the times these were geared by personal aspirations. Individual motives and prospects of profit meant that these official-cum-merchants ('portfolio-capitalists') could build convenient partnerships with certain VOC officials for ousting common enemies or clash with them as competing rivals in de commercial sector. Conversely, both of them used their institutional garb for fighting personal battles, destroying each other's forts and factories. The large potentates like Shaista Khan had their own stakes in the trade in cotton, silk and other commodities from Bengal.[91] Prince Azim-ush-Shan who had earlier been the *subahdar* of Bengal and Bihar even wanted to monopolise all imports in the province entitling it as *Sauda-i-Khas-o-aam*.[92] Thus, when the commercial enterprises of these Mughal personalities functioning at a regional level faced chances of being hampered because of the English and Dutch Companies' attempts at monopoly, there was understandably desperation to threaten each other.

But it was certainly the voices of these locals as the Mughal *subahdars* that found free vent in the sources. They were not only heard but also authoritatively asserted. Shaista Khan, for example, wrote a letter to the Dutch official Van Reede who had been investigating the factories of Bengal in 1686 about the Company servants' misbehaviour and the damage that they had caused through illegal trade with the local merchants (*gomostas*) in the villages leased out to them.[93] Mir Jumla, earlier in 1661 had the power to lobby a group of local silk merchants for forwarding a petition to the Mughal emperor against the Company's duty free trade in Kasimbazaar. It led to a restraint being put on the amount of raw silk that the Company could henceforth procure from there.[94] There are plenty of such other examples in the VOC archives which show that these officials communicated directly with the Dutch and did not hesitate to enter into open conflicts as they lacked neither military resources nor finance. This legal case simply confirms that idea of these local voices playing a dominant role in determining the VOC's fate in Bengal.

In the perceptions of these locals, the Dutch were relegated to another extreme that was different from the perception emerging out of the *Mangalkavya* literature. Here, the Mughal officials perceived the Dutch simply as merchants under their subjugation. They had the required power and resources to keep the European Companies under strict vigilance and be able to take action in case of non-compliance with their orders. It is a relation that seemed rough and not always easy going even though elite officials

[90] See for cases of the VOC entering into legal dialogues with the local authorities to claim areas, Clulow, 'The Art of Claiming', p. 37.
[91] Chandra, *Essays*, p. 233.
[92] Ibid., p. 234.
[93] NA, VOC 1422, f. 1255r.-1256r.
[94] Prakash, 'The Dutch East India Company', p. 277.

had to communicate regularly with the Dutch. At least in the minds of these locals, the Dutch rarely escalated to the core political level. This does not discount the attempts on the part of the Dutch in harbouring territorial acquisitions, but indicates that the Mughal officials did not consider them competent enough or legitimate enough to share political power. It was more of an uptight atmosphere where commerce backed by military manoeuvrings could at times lead to open conflicts and at other times, remain dormant in the form of calculated collaborations. By all means therefore, one can conclude that the perception of the Company and its officials in Bengal differed substantially among these locals as compared to the ordinary villagers and small brokers or the intermediate landlords and kings rooted at different social ranks and positions.

## Conclusion

This essay has tried to contribute to the extensive historiography of the Indo-European relations by focusing on the VOC in seventeenth century Bengal. Challenging the connotations of 'conflict', 'collaboration' or 'competition', it has proposed an alternative dimension that reveals the layered and nuanced aspect of these relations. In the process, perceptions of locals coming from different social backgrounds and the motives behind their interactions with the Dutch have been unfurled. Consequently, the multiple angles of local insight into the Dutch presence in Bengal have also been studied. The local texts patronised by several kinglets and non-Islamic elites portrayed a stereotypical image of the Europeans including the Dutch as the powerful overlords of the tropical waters. However, on closer analysis one finds that such depictions originated later in the eighteenth century conditioned by the changing political situation. In the seventeenth century, the nature of their contact with the Dutch or other Europeans were still loose and not serious in the context of the strong, legitimate Mughal presence.

This perception contrasted starkly with the locals depicted in the painting of Schuylenburgh. These locals as workers and small brokers were shown to be much closer in their dealings with the Company and its servants. It was likely that their perception would have offered a stronger overview of the Dutch. The legal case in the end provided a very different perspective of those locals who were powerful enough as political actors to manipulate the Dutch. People like the subahdar, the faujdar, the diwan or karori held substantial powers to dictate the Company's course and curb or tickle the ambitions of its officials as per their convenience. Their views on the Dutch was therefore not high, but they needed to keep contact with the Company's men having substantial military and naval resources. Both sides were compelled to interact with each other for their individual interests, albeit at the cost of frequent conflicts and clash of power.

The dynamics of Indo-Dutch relations in Bengal was thus shaped by personal forces complicated with layers of diverse interests, positions and powers. The insight of the local population of Bengal consequently varied with their diverse social ranks, outlook and profit motives. Whether the locals saw the Dutch as competitors or fierce rivals, whether they sniffed trouble in the air or experienced them as a group of potential rulers; whether they understood and adapted each other's modes of expression or distanced themselves; whether they got a taste of being with each other and working together or had frequent frictions and contempt; and whether they affected each other's' lives or loathed the very space they shared was all a matter of how a bunch of eyes, ears, tongues, mouths and hearts reacted from varied stances. In that large mosaic of people, goods, texts and other objects floating around, these diversities thriving on personal aspirations contributed to Bengal's vitality as a nodal point in the Indian Ocean trading network. Subsequently not only the Dutch but also the Persians, the Chinese, the Armenians, the French, the English and everyone else came to be perceived in the way they tried to fit themselves in the existent picture. And of course, the way they came to be received by the different local groups varied, depending on the wavelengths of their power and position in Bengal.
